# Antioxidant-Enhanced Alginate Beads for Stabilizing Rapeseed Oil: Utilizing Extracts from Post-Distillation Waste Residues of Rosemary

**DOI:** 10.3390/foods13132142

**Published:** 2024-07-05

**Authors:** Petroula Tsitlakidou, Despina Kamplioni, Anastasia Kyriakoudi, Maria Irakli, Costas G. Biliaderis, Ioannis Mourtzinos

**Affiliations:** 1 Laboratory of Food Chemistry and Biochemistry, Department of Food Science and Technology, School of Agriculture, Faculty of Agriculture, Forestry and Natural Environment, Aristotle University of Thessaloniki (AUTH), 54124 Thessaloniki, Greece; ptsitlak@agro.auth.gr (P.T.); despwkamb@gmail.com (D.K.); ankyria@agro.auth.gr (A.K.); biliader@agro.auth.gr (C.G.B.); 2 Institute of Plant Breeding and Genetic Resources, Hellenic Agricultural Organization—Demeter, P.O. Box 60458, Thermi, 57001 Thessaloniki, Greece; mirakli@elgo.gr

**Keywords:** alginate, emulsion gels, rosemary distillate residues, rapeseed oil, encapsulation, oxidation stability

## Abstract

An eco-friendly extraction process of polyphenols from conventional dried rosemary tissues and post-distillation waste residues was applied using β-cyclodextrin as a co-solvent. The aqueous extracts were characterized by measuring the total phenolic content, and their phenolic compounds were identified and quantified by LC-MS. Sodium alginate solutions (2% *w*/*w*) with/without incorporation of rosemary aqueous extracts were prepared and used for the preparation of O/W emulsions containing 20% rapeseed oil and an 80% water phase. Hydrogel beads were then stored at 20 °C for 28 days. The quality of encapsulated oil during storage was evaluated by measurements of the peroxide value, p-anisidine value, free fatty acids, total oxidation value, and fatty acid composition, whilst the aqueous phase of the beads was analyzed for its total extractable phenolic content (TEPC). The experimental findings indicate that the incorporation of aqueous extracts from post-distillation rosemary residues in emulsion-filled hydrogel beads resulted in the lowest level of oxidation products in the encapsulated rapeseed oil (PV = 10.61 ± 0.02 meq/Kg oil, p-AnV = 4.41 ± 0.09, and FFA = 0.14 ± 0.00, expressed as % oleic acid content), indicating an acceptable oil quality until the end of the storage period.

## 1. Introduction

Consumer awareness towards healthier diets and the strong link between nutrition and health have triggered an increase in the production and commercialization of foods with increasing amounts of polyunsaturated fats [1] as well as the substitution of “chemical additives” with “natural” alternatives in food formulations. As a result, aromatic and medicinal plants (AMPs) have been gaining a growing interest as a source of natural bioactive compounds with potential uses in the food industry; moreover, aromatic plants and mainly their essential oils and extracts have been used for centuries as flavouring ingredients and for food preservation purposes because of their well-documented antimicrobial and antioxidant properties [2].

*Rosemary officinalis* L. (rosemary) is a medicinal and aromatic plant, and several studies have revealed its distinguishing polyphenolic diversity and antioxidative potency; the main bioactive ingredients of this plant are carnosic acid, carnosol, and rosmarinic acid [3,4]. Moreover, rosemary is heavily exploited in the production of essential oil (e.g., via hydrodistillation), generating high amounts of solid residues due to the low yield of essential oil [5]. The production of essential oil rarely yields more than 0.3–2.5% *w*/*w* of dry plant in the case of rosemary [6], whilst the essential oil yield of AMPs generally varies between 0.5 and 8% *w*/*w* of dry biomass [7].

EOs are natural, complex mixtures of volatile compounds, which are formed in the plant material as secondary plant metabolites. Terpenoids are the most important chemical group of EOs and are compounds formed by the conjunction of isoprene units. As a rule, isoprene units are joined in one direction, coupling head-to-tail, i.e., terpenoids contain in their structure several five-carbon atom entities. They may be hydrocarbons, or oxygenated derivatives (alcohols, aldehydes, ketones, or acids) or reaction products (esters and ethers). Additionally, non-terpenic compounds generated via the phenyl propanoid pathway are EO constituents, i.e., eugenol, cinnamaldehyde, safrole, etc. [8]. Oxygenated compounds are mostly responsible for flavouring properties, demonstrating a high odour intensity score [9]. The chemical composition of EOs is an important criterion in their utilization for several uses and a valuable quality marker for marketing, thus contributing to their potential valorization. Several factors (extraction techniques, plant raw material varying in composition due to physiological, biological, genetic, and environmental conditions) influence EO composition, and the major constituents of rosemary’s essential oil are 1,8-cineole (43.77–88.9%), camphor (2.4–12.53%), α-pinene (2.7–11.51%), and β-pinene (0–8.16%) [10].

The current tendency for sustainable development, involving cyclic economy principles, largely focuses on the valorization of agro-industrial by-products and the recovery of valuable compounds with bioactivities, mostly related to their antioxidant and other human health-promoting effects. Previous studies suggested the potential exploitation of the solid residues from the hydrodistillation of rosemary as a valuable source of polyphenols with antioxidant activities [11,12,13]. Whilst the main focus of those studies was the recovery of phenolic compounds and the techniques applied for their extraction, there have been no studies on the use of phenolic extracts obtained from rosemary solid waste streams of the EOs industry in food applications.

The potential exploitation of bioactive compounds present in distillation residuals by extraction of phytochemicals and further conversion into value-added products could address a number of Sustainable Development Goals (SDGs) laid out in the 2030 Agenda for Sustainable Development [14]. The 17 Sustainable Development Goals (SDGs) are intended to address unfulfilled issues such as extreme poverty, inequality, social injustice, and protection of the environment by 2030 [15]. The development of eco-friendly extraction techniques and valorization of solid waste streams of the EOs industry could promote the achievement of SDG 9 and SDG 12, i.e., to build resilient infrastructure, promote inclusive and sustainable industrialization, and foster innovation (SDG 9), as well as to ensure sustainable consumption and production patterns (SDG 12).

Sodium alginate is a renewable, biodegradable, biocompatible, and non-toxic polysaccharide extracted from brown seaweed. Alginates, being ionic polysaccharides, are capable of forming hydrogels (Ca^2+^-mediated) in various morphologies (e.g., beads, fibres) ideal for functioning as carriers of bioactive compounds with the additional benefit of controlled release under the typical pH conditions encountered in the human GI tract [16]. They are linear polymers composed of 1,4-linked β-D-mannuronic acid (M) and 1,4 α-L-guluronic acid (G) anhydro residues interlinked in homogeneous (poly-G, poly-M) or heterogeneous (mixed MG) block-like patterns on the chains [17]. The relative amounts of α-L-guluronic acid (G) and β-D-mannuronic acid (M) groups in the alginate chains (i.e., the G/M ratio) determine the permeability and physicochemical properties of the ionotropic hydrogels formed with these polysaccharides [18]. The G/M ratio varies depending on their natural source (seaweed), location in the plant, geographical location, and the extraction process adopted [19], i.e., different types of alginates are commercially available with varying structural features depending on the seaweed species.

Besides biocompatibility, biodegradability, and environmental friendliness, alginate hydrogels are cost-effective, simple to produce [20], and have relatively low capital and operating costs [21]. Alginates demonstrate great capability to be cross-linked with other natural or non-natural materials and build a wide range of alginate-based systems such as hydrogels, emulsions, emulsion-filled hydrogels, liposomes, edible films, and aerogel [22]. In terms of food applications, alginates can be used as viscosifiers or gelling agents as well as an edible coating material to extend the shelf-life of foods by maintaining the moisture, flavour, and reducing fat oxidation [23]. Another emerging application of alginate in the food industry involves creating emulsion-filled hydrogels as carriers of health-promoting ingredients [24] or fat replacers due to their thermal stability [22], enabling the development of “clean label” products and the use of nutritional claims. Moreover, recent studies on double emulsions fortified with olive leaf extract [25] and emulsion gels containing a high content of unsaturated fatty acids and tannins [26]—as functional fat replacers in meat systems—suggested that the development of those food systems is a promising strategy for enhancing antioxidant properties and, thus, preventing lipid oxidation in those reformulated systems. In this context, encapsulation of olive oil [27,28], ginger oil [29], linseed oil [30], and sunflower oil [31] by means of alginate gelation provided enhanced stability of the respective oil against oxidation. Yet, little information is available on the encapsulation of flavours in sodium alginate beads; in this respect, previously published works demonstrated that encapsulation of flavour components [32] and complex mixtures [33] in alginate hydrogels improved the stability and retention, and permitted the controlled-release of flavours during thermal processing, which are important properties affecting the flavour loss in final products.

In view of the strong antioxidant potency of rosemary extracts and the ability of alginate to form emulsion-filled hydrogels, the incorporation of antioxidant compounds in the aqueous phase of an emulsion and the use of rapeseed oil with high content of oleic and linoleic acids in the oil phase may serve as a dual strategic approach in formulating emulsion systems: first, to prevent the oxidative deterioration of the oil phase and, thus, enhancing its nutritional value, and second, to optimize the presence of bioactive compounds in the dispersion in which polyphenols extracted from rosemary will be included as an ingredient. Therefore, the objective of the present study was to encapsulate in alginate beads rapeseed oil (in an O/W emulsion) loaded with polyphenols extracted from rosemary using two different streams of plant materials (tissues from the conventional raw plant material of rosemary and post-distillation waste residues) and evaluate the effect of the recovered antioxidants on storage stability of the oil and the retention of the extracted polyphenols during storage. The information obtained in this study might be also useful for the development of novel encapsulation approaches by valorizing post-distillation waste residues of aromatic plants while working towards the “zero-waste” biorefinery concept.

## 2. Materials and Methods

### 2.1. Plant Material

Dry-rubbed rosemary leaves (*Rosmarinus officinalis* L.) were obtained from Physis Ingredients; the plants were organically cultivated in the region of Serres, in northern Greece, and shade-dried after harvesting. Cold-pressed rapeseed oil (*Brassica campestris* seed oil) was purchased from AOT Organic products (Wiggensbach, Germany).

### 2.2. Chemicals, Reagents, and Solvents

Alginic acid in the form of sodium salt with low viscosity (viscosity: 66 mPas at 1% *w*/*w* in aq. solution) was obtained from Thermo Fisher Scientific (Athens, Greece) and β-cyclodextrin (CAS No: 7585-39-9, MW: 1134.99 g/mol) was sourced from TCI-Tokyo Chemical Industry Co., Ltd., Tokyo, Japan. Tween 80- Polyoxyethylene sorbitan monooleate (100%, CAS No: 9005-66-6) was purchased from Duchefa Biochemie B.V. (Haarlem, Netherlands). Calcium chloride (>98%, CAS No: 10043-52-4), anhydrous citric acid (>99.5%, CAS No: 5949-29-1), methanol (>99%), hexane (>95%), acetic acid (99–100%), chloroform (>99%), and sodium thiosulfate, Na_2_S_2_O_3_ (>98%, CAS No: 10102-17-7), were obtained from Chem-Lab NV (Zedelgem, Belgium). Corn starch (CAS No: 9005-25-8) and potassium iodide (≥99%, CAS No: 7681-11-0, MW: 166 g/mol) were purchased from Sigma Aldrich (Taufkirchen, Germany).

### 2.3. Isolation of Rosemary Essential Oil

Hydrodistillations of rosemary leaves were conducted at a laboratory scale according to the 10th edition of the European Pharmacopoeia [34]. Specifically, 25 g of dried rosemary leaves was added in a 500 mL round bottom flask, 300 mL of distilled water was added, and then it was submitted to water distillation for 2 h using a Clevenger apparatus. The distilled essential oil was collected in a vial, which was hermetically sealed and stored at −18 °C until further use.

### 2.4. Preparation of Post-Distillation Rosemary Residues

**Figure 1:**
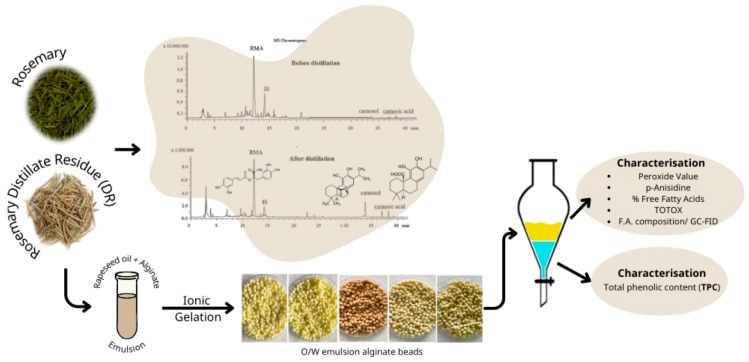
Flow diagram describing the experimental procedure.

For the preparation of post-distillation rosemary residues, the hot water/rosemary leaves mixture was removed from the round bottom flask immediately after the end of hydrodistillation and the residues of rosemary leaves were collected. The residual leaves were dried in an air drier for 24 h at 40 °C until the weight of rosemary biomass was stable (~10% moisture content). The dried material was subsequently milled to pass through a 2.0 mm sieve and stored in heat-sealed bags at ambient temperature until further analysis. A flow diagram describing the experimental procedure is displayed in Figure 1.

### 2.5. Green Extraction using Aqueous Solutions of β-Cyclodextrin

Samples of dried and ground post-distillation rosemary solid residues were extracted with an aqueous solution of β-cyclodextrin (0.925% *w*/*v*) at a liquid-to-solid ratio: L/S = 8 mL/g. The extraction was held at 50 °C for 124 min and under constant stirring at 600 rpm. The experimental conditions for extraction were chosen based on previous research with slight modifications [13,35]. Following extraction, the extracts were obtained by filtration through filter paper (Whatman No. 1), removing the main solid biomass, and subsequently centrifuged at 6000× *g* rpm for 10 min using a bench centrifuge (Hettich Universal 32, DJB Labcare Ltd., Newport Pagnell, UK). The clarified supernatants were placed in a stoppered glass bottle and stored at −18 °C until analysis.

### 2.6. Determination of Total Phenolic Content (TPC) in Aqueous Extracts

The total phenolic content of the samples was determined according to a published protocol [36] using the Folin–Ciocalteu methodology and gallic acid as the standard; the results were expressed as milligrams of gallic acid equivalents per g sample on a dry weight basis (mg GAE/g dw) using a calibration curve. Measurements were performed in triplicate.

### 2.7. LC-MS Analysis of Aqueous Extracts

LC-MS analysis was performed using a Shimadzu Nexera HPLC system (Shimadzu, Kyoto, Japan) equipped with a diode array detector (DAD) and a single quadrupole mass spectrometer, combined with an electrospray ionization source [12]. The separation of phenolic compounds was achieved on a Poroshell 120 EC-C18 column (4.6 × 150 mm, 4-micrometre particle size), thermostatted at 35 °C, with mobile phase A (0.1% aqueous formic acid) and B (acetonitrile) at a flow rate of 0.5 mL/min, following a 45-min linear gradient elution as follows: 15–25% B, 0–5 min; 25–35% B, 5–10 min; 35–60% B, 10–28 min; 60–100% B, 28–35 min; 100–15% B, 35.01–40 min; followed by an isocratic elution (15% B) until 45 min. The mass spectrometry was carried out in negative ion mode and the quantitative determination of target compounds in the phenolic extracts was performed in the selected ion monitoring (SIM) mode, using calibration curves of the respective standard solutions at five concentration levels within the linear range of 0.01 to 10 μg/mL for rosmarinic acid, and 0.05 to 200 μg/mL for carnosol and carnosic acid. Data acquisition and processing were performed using the Lab Solutions LC-MS software version 5.97.SP1 (Shimadzu, Kyoto, Japan). Analyses were performed in triplicate and the results were expressed as mg per g of extract.

### 2.8. Preparation and Storage of O/W Emulsion Alginate Beads

#### 2.8.1. Preparation of O/W Emulsions with Alginate

For preparation of the aqueous continuous phase, sodium alginate (2% wt) was dissolved in the two types of aqueous rosemary extracts (aqueous extract of dry rosemary and aqueous extract of post-distillation rosemary waste residues) as well as in deionized water in the case of control samples. Both aqueous extracts and deionized water were previously adjusted at pH 5.00 by means of a dilute citric acid solution (pH = 2.00). The solubilization of sodium alginate was carried out under magnetic stirring at 500 rpm at room temperature for 2–3 h, and once dissolution of the alginate powder particles was achieved, the alginate solutions were stored overnight at below 8 °C prior to their use for the preparation of the emulsions. For preparation of O/W emulsions, rapeseed oil was firstly mixed with a specified amount of Tween 80, and then the water phase (alginate alone or alginate dissolved in aqueous rosemary extracts) was slowly added and sheared at 1000 rpm for 20 min. The final emulsion system consisted of 80% aqueous phase (containing 2% sodium alginate and 0.5% Tween 80) and 20% rapeseed oil (in the presence or absence of rosemary essential oil incorporated in the rapeseed oil at a level of 0.1% *w*/*w*). Table 1 illustrates all the formulations used for preparing the O/W emulsions. The emulsions were freshly made each time and no phase separation was visually observed after placing them into cylinders of 100 mL when left for at least 1 h.

#### 2.8.2. Preparation and Storage of Alginate Beads

For producing the emulsion alginate beads, the formulated O/W emulsions were placed into a square mould (9 cm × 9 cm × 6 cm) fitted with a frame consisting of 100 pores of 0.3-centimetre diameter (as illustrated in Figure A2). Ionic gelation (cross-linking) was elaborated by dropping the emulsion droplets into a 2.0% (*w*/*w*) CaCl_2_·2H_2_O solution. The distance between the mould (generating the emulsion droplets) and the surface of the CaCl_2_ solution was 6 cm. The immersed emulsion droplets in the CaCl_2_ solution were allowed to gellify (“hardened” via ionic exchange for the alginate chain cross-linking) for 30 min without any stirring. After gelation, the resultant beads were rinsed with deionized water, dried by an absorbent paper towel, placed in plastic Petri dishes, and stored in a cupboard at ambient temperature (20 ± 2.0 °C).

### 2.9. Oxidative Stability Study

To study the ability of the different emulsion formulations to protect rapeseed oil from oxidation, each emulsion alginate bead preparation was placed in a Petri dish, whereas a small amount of free, non-encapsulated rapeseed oil was put in another dish as the control preparation (RPSO). All dishes were stored at 20 °C and away from sunlight for 28 days to carry out the oxidative stability study. All chemical assays were performed at the beginning of storage (Day 0), and periodically after 7 (Day 7), 15 (Day 15), and 28 (Day 28) days of storage. Analysis of the samples was performed in quadruplicate at every sampling time interval in the oxidative stability study (*n* = 4).

#### 2.9.1. Extraction of Rapeseed Oil from the Alginate Beads

The rupture of emulsion alginate beads was managed according to Flaminii et al.’s method (2020) [37] with slight modifications. At every sampling time interval, a Petri dish from all the different sample preparations was used for the analysis. Briefly, a 10% (*w*/*v*) sodium citrate solution was prepared and mixed with the alginate beads at a weight ratio of 5:1. The dispersions were left for 3 h at ambient temperature until the beads were greatly deformed. Extraction of rapeseed oil was then performed following the method reported by Sun-Waterhouse et al. (2011) [28] and Atencio et al. (2020) [29] with slight modifications. The dispersion of the deformed alginate beads was mixed with 100 mL of methanol and slightly shaken until aggregation of the polysaccharide matrix was noted, and the mixture was transferred into a separatory funnel. Hexane (100 mL) was then added, and the mixture was vigorously agitated to enable the transfer of oil into the hexane top layer, which was collected. The extraction steps were repeated three to four times, with the obtained hexane layers being combined, and the hexane was finally removed using a rotary evaporator (Rotavapor R114, Waterbath B480, Buchi, Flawil, Switzerland). Small amounts of the remaining hexane evaporated from the oil under the flow of a nitrogen gas stream.

#### 2.9.2. Peroxide Value (PV) Determination

The PVs of all extracted oil samples were determined according to the AOCS Official Method [38], with all reagents at 10% of the amount recommended for the standard protocol [39] and expressed as peroxide milliequivalents per kg of oil. Oil (0.5 g) was mixed in a stoppered flask with acetic acid-chloroform solution (3 mL, 3:2 *v*/*v*). Then, saturated potassium iodide KI solution (50 μL) was added, and the mixture was shaken vigorously and left to stand for 1 min. An aliquot (3 mL) of distilled water was subsequently added, and the mixture was titrated with 0.001 N standardized sodium thiosulphate solution (for liberated iodine) until the light yellow colour almost disappeared; a small portion (0.2 mL) of a starch solution (1% *w*/*v*) was used as the indicator, with the titration being continued until the blue colour derived from the iodine disappeared. A blank sample as a control reagent was set up and analyzed similarly according to all the previous steps. The PV was obtained from the formula:

(1) $$PV=\frac{1000\left(V-Vo\right)\;C}{m}$$

where V = volume of Na_2_S_2_O_3_ titrant of sample (mL); Vo = volume of Na_2_S_2_O_3_ titrant of blank (mL); C = normality of Na_2_S_2_O_3_ (moles/L); m = sample mass (g).

#### 2.9.3. p-Anisidine Value (p-AnV) Determination

The determination of p-AnVs (a measure of aldehyde levels in oils) was performed according to the AOCS Official Method [40]. The procedure was carried out in such a way to minimize the exposure of all preparations to sunlight. Briefly, p-anisidine solution was prepared by mixing 0.25 g of the reagent with 100 mL glacial acetic acid and the solution was kept in the dark. Approximately 0.5 g of the extracted oil samples was first dissolved in iso-octane (25 mL). The solution was allowed to stand for 10 min and the absorbance was measured (UV-1800, UV/Visible scanning spectrophotometer, SHIMADZU, Kyoto, Japan) at 350 nm and denoted as A0, using isooctane as the blank solution (A2). Then, an aliquot (5 mL) of the oil solution was mixed with 1 mL of the 0.25% p-AnV reagent. After 10 min, the absorbance of the reacted mixture (yellowish reaction products) was read at 350 nm and denoted as A1. The p-AnV of the sample was then calculated using the following equation:

(2) $$p\text{-}AnV=\frac{25\times \left[1.2\left(A_{1}-A_{2}\right)-A_{0}\right]}{m}$$

where m = sample mass (g).

#### 2.9.4. TOTOX (Total Oxidation) Value (TV) Determination

TVs were calculated from the sum of twice the peroxide value (PV) and the anisidine value (p-AnV) as follows [28,41]:

(3) Totox value=2PV+(p-AnV)

#### 2.9.5. Free Fatty Acids (FFA) determination

FFAs of the extracted oil samples were determined using the direct titration method of AOCS [42] with slight modifications. Neutralized alcohol was prepared with absolute ethanol (0.625 mL), oil (0.1 mL), and 1% (*w*/*v*) phenolphthalein indicator (50 μL) in a conical flask. The flask was then placed in a water bath (60 °C) until warmed. Sufficient NaOH (0.005 M) was added to produce a faint permanent pink colour. Extracted oil samples were weighed (0.7 g), dissolved into the neutralized alcohol, and titrated with the NaOH solution until a faint permanent pink colour appeared (phenolphthalein-determined endpoint). The FFA content was expressed as oleic acid (percentage):

(4) $$\%FFA\left(as\;oleic\;acid\right)=\left(\frac{V_{NaOH}\times C_{NaOH}\times 282.46}{w}\right)\times 100$$

where V = volume of NaOH titrant (ml); C = molarity of NaOH titrant (mol/1000 mL); 282.46 = MW of oleic acid (g/mol); W = sample mass (g).

#### 2.9.6. Determination of the Total Extractable Phenolic Content (TEPC)

The total phenolic content of the aqueous phase, obtained after rupture of the emulsion alginate beads and removal of oil, was determined according to the method described in Section 2.5. The results were expressed as milligrams of gallic acid equivalents per L of sample (mg GAE/L) using a calibration curve. Measurements were performed in triplicate.

#### 2.9.7. Fatty Acid Profile/Composition Analyses

For the determination of the fatty acid profile, transesterification was carried out for the extracted oil samples to proceed with gas chromatographic analysis. In particular, 0.1 g of extracted lipids was transferred in a test tube with a screw cap and 2 mL of *n*-hexane was added, followed by addition of 0.2 mL 2.0 M methanolic solution of potassium hydroxide for preparation of the fatty acid methyl esters (FAMEs). The mixture was vortexed for 1 min and was left to settle until the upper phase that contains the FAMEs became transparent. The phase that contained the methyl esters was collected, filtered (0.45 µm PTFE hydrophobic filters), and analyzed by a gas chromatograph (TRACE GC 2000 Series, Thermo Quest CE Instruments, Rodano (MI), Italy) with a flame ionization detector (FID) equipped with an autosampler (TRIPLUS AS Thermo Quest CE Instruments). FAMEs were analyzed on a BPX70 GC capillary column (30 m length, 0.32 mm, i.d., 0.25 μm film thickness, SGE Analytical Science, Stockbridge, GA, USA). Helium was the carrier gas at a flow rate of 2.0 mL/min. The injector port and detector temperature were maintained at 250 °C. The split ratio was 1:20. The column oven was initially set at 46 °C for 2 min; then, the temperature was raised to 130 °C at a rate of 50 °C/min and maintained there for 10 min; then increased to 175 °C at 2 °C/min and maintained at that temperature for 2 min; then increased to 200 °C at 3 °C/min and maintained at that temperature for 3.5 min; before raising finally the temperature to a plateau of 240 °C at a rate of 10 °C/min and maintaining it at this level for 5 min, i.e., the total run time was 60 min. Identification of FAMEs was carried out by comparing the retention times (RT) with those of a standard mixture (AccuStandard, New Haven, CT, USA) containing 37 fatty acids and analyzed under the same chromatographic conditions [43]. Chromatograms were acquired and processed with the aid of the Chrom Quest 5.0 software (ver. 3.2.1, Thermo Separation Products, Thermo Scientific, West Palm Beach, FL, USA).

### 2.10. Statistical Analysis

Basic statistical parameters (average and standard deviations) were calculated by Microsoft Excel 2016. At every sampling time interval in the oxidative stability study, the obtained results were analyzed by one-way ANOVA with Tukey’s ad hoc test by using Minitab 19 to determine significant differences (*p* < 0.05) between the mean values obtained for the measured parameters of beads with different compositions.

## 3. Results

### 3.1. Total Phenolic Content (TPC) in Aqueous Extracts

**Figure A1:**
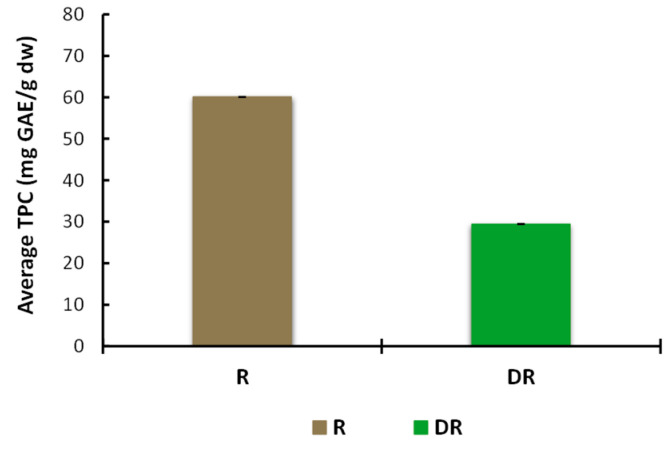
Total phenolic content (TPC) in aqueous extracts of dried ground raw rosemary (R) and dried ground post-distillation residue (DR). Data are expressed as means ± standard deviation; measurements were performed in triplicate.

**Figure A2:**
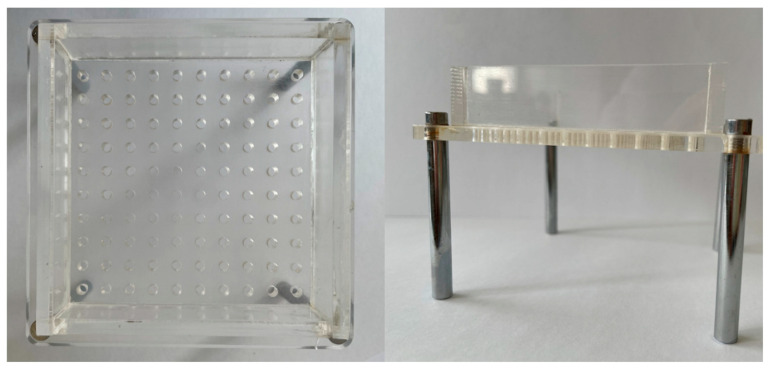
Illustration of the mould used for the preparation of the O/W emulsion alginate beads.

The concentration of phenolic compounds in the aqueous extract of undistilled rosemary (R) was two times higher than that of the rosemary post-distillate residues (DR) (Figure A1). These levels are in agreement with the findings of Christaki et al. (2022) [13], who reported a higher phenolic concentration (expressed in mg GAE/g extract) in aqueous methanolic (70%) extracts obtained by ultrasound treatment of the post-distillation (EO hydrodistillation) plant materials of rosemary, sage, and spearmint compared with the extracts of the respective undistilled aromatic medicinal plants. Moreover, they reported that hydrodistillation showed the greatest negative impact on the recovery of TPC from solid residues, compared with two other methods studied, i.e., steam distillation and microwave-assisted hydrodistillation. The considerable losses of bioactive compounds from the solid residues following hydrodistillation have been previously attributed to the extended distillation time, leading to the destruction of thermolabile compounds, as well as the nature of some compounds that are water-extractable solubles and, thus, partition in the residual wastewater phase [44,45].

Besides the distillation method, the extraction conditions and the method applied to treat post-distillation rosemary waste residues can also affect the recovery yield and the composition of the extracts. Among the extraction methods, accelerated solvent extraction at elevated temperature and pressure [46], ultrasound-assisted extraction [12,13], and solid–liquid extraction with organic solvents [47] have been previously examined to exploit the rosemary solid residues as a source of phenolics after removal of the essential oil; the reported TPC values in the present study are in agreement with the data available in the scientific literature. Regardless of the applied extraction method and conditions, rosemary solid residues could be generally considered as a valuable post-distillation waste by-product that could be valorized for the recovery of important bioactive compounds.

### 3.2. LC-MS Analysis

**Figure 2:**
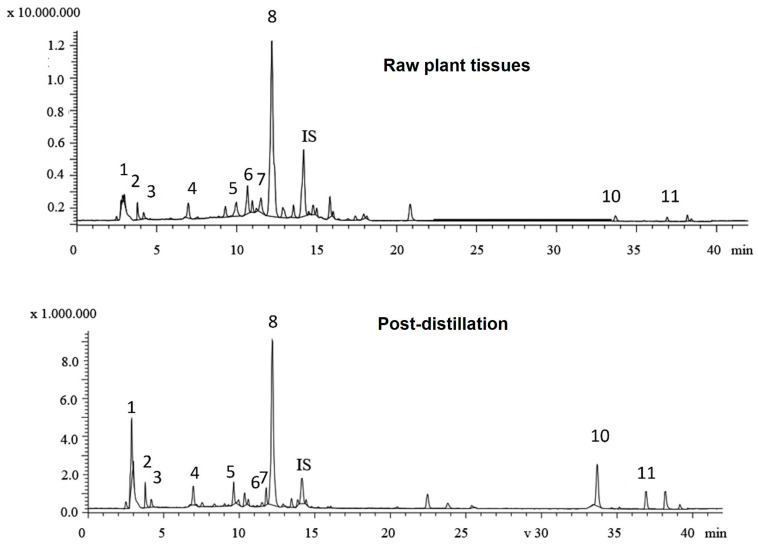
Total ion chromatograms of the phenolic compounds in aqueous extracts from raw plant tissues and post-distillation residues of rosemary. (1: quinic acid; 2: neochlorogenic acid; 3: vicenin-2; 4: caffeic acid; 5: luteolin-7-O-rutinoside; 6: apigenin-7-O-glucoside; 7: hesperidin; 8: rosmarinic acid; 9: salvianolic acid B; 10: carnosol; and 11: carnosic acid).

LC-MS analysis was performed to identify the phenolic compounds present in two typical rosemary aqueous extracts (raw plant tissues vs. post-distillation plant residues, Figure 2), as the Folin–Ciocalteu assay only gives an estimate of the total phenolic functionalities and cannot be directly related to compositional analysis data. Table 2 presents the content of the major phenolics identified in the aqueous extracts. In total, eleven phenolic compounds were identified and quantified, including five phenolic acids, four flavonoids, and two phenolic diterpenes in both of the aqueous extracts of rosemary tissues. The major phenolic acids identified were rosmarinic acid, salvianolic acid isomer B, and quinic acid, whilst minor peaks of caffeic acid and neochlorogenic acid were also found in both aqueous extracts. The rosmarinic acid was the most dominant compound, constituting more than 82% of the total quantified phenolic acids in both samples. Rosmarinic acid was previously described as the most abundant compound in rosemary infusions [48] and, in fact, is considered a “family marker” for the *Lamiaceae* family [49]. The aqueous extract of undistilled (raw) rosemary (R) contained a higher total content of phenolic acids (65.2 ± 0.38 mg/g) than the aqueous extract from the post-distillation rosemary waste (DR) residues (42.73 ± 0.28 mg/g). This observation can be largely attributed to differences in rosmarinic acid content between the two extracts as the initial (R extract) rosmarinic acid content of 56.6 mg/g decreased significantly to 35.3 mg/g in the rosemary distillate extract (DR). Previous studies on rosmarinic acid content in extracts obtained prior to and after the hydro- and steam-distillation of *Rosmarinus officinalis* L. have reported similar findings [13,50]. The decrease in the recovery of rosmarinic acid can be explained by its thermal degradation during distillation and its water solubility as it has been previously shown that substantial amounts of rosmarinic acid are detected in the water phase during the hydrodistillation of *Rosmarinus officinalis* L. residues [50]. Nevertheless, considerable amounts of rosmarinic acid were present in both extracts; in fact, it was the most abundant compound among the identified polyphenols, thus, it can be considered as a relatively stable compound [44,51].

On the other hand, the total content of phenolic diterpenes in the aqueous extract of post-distillation rosemary residues (DR) was four times higher than that of the undistilled rosemary (R). There is a large body of literature stating that the diterpenoids, carnosol, and carnosic acid are the most important active components of rosemary extracts [3,4,51,52,53]. Oreopoulou et al. (2018) [54] reported that after alkaline extraction of hydrodistillation residues, *Rosmarinus officinalis* demonstrated the highest antioxidant activity in sunflower oil, and this was attributed to the presence of carnosic acid. In this context, the concentration of carnosic acid was shown to be highly correlated with the oxidative stability of the sunflower oil [55]. Both carnosic acid and carnosol have been proposed as compounds comprising over 90% of the antioxidant potential of rosemary extracts [54]. The aqueous extract of raw rosemary contained 0.58 mg/g of carnosol and 2.51 mg/g of carnosic acid, whilst the amounts found in the extract of post-distillate rosemary residues were 5.67 mg/g and 8.22 mg/g, respectively. The amount of carnosol present in both aqueous extracts is dependent on the applied extraction process; carnosol, as well as rosmanol, are generated by oxidative degradation of carnosic acid and thus as native constituents of unprocessed (raw) rosemary leaves exist in trace or very small amounts [50]. Moreover, carnosol content may increase at the expense of carnosic acid when the plant biomass is subjected to drying, heating, storage, extraction, and distillation [4]. Non-polar solvents were shown to extract considerably higher amounts of phenolic diterpenes [6,12,13] in comparison to the levels obtained by aqueous media. Apparently, only small amounts of carnosol have been found in the hydrodistillation water following the distillation of rosemary leaves, presumably due to its high hydrophobicity [50]. On the other hand, organic solvents suitable for the extraction of less-polar compounds are not consumer/environment-friendly, and as a result, more “green solvents”—often combined with innovative extraction methods—are preferable nowadays for isolation purposes. In the current study, the use of β-cyclodextrin in water as a “green” extraction medium could have enhanced the extraction efficiency of the aforementioned polyphenols due to the formation of inclusion complexes with the cyclic dextrin, as this has been previously reported [56,57,58].

### 3.3. Protection of Oil against Oxidation

The evolution of oxidative deterioration of rapeseed oil with time was determined in either free, non-encapsulated rapeseed oil (RPSO) or in encapsulated forms of various emulsified preparations (O/W oil/aqueous phase, 20/80 *w*/*w*), i.e., rapeseed oil alone (control), rapeseed oil containing rosemary essential oil (EO), rapeseed oil with aqueous phenolic extract from raw rosemary plant material (AER), rapeseed oil with aqueous phenolic extract from the post-distillation of rosemary waste residue (AERDR), and rapeseed oil with a combination of rosemary essential oil and the aqueous phenolic extract from the post-distillation of rosemary waste residue (AERDR/EO). The composition of the various formulations of encapsulated rapeseed oil emulsions in the alginate beads is presented in Table 1.

#### 3.3.1. Peroxide Values

**Figure 3:**
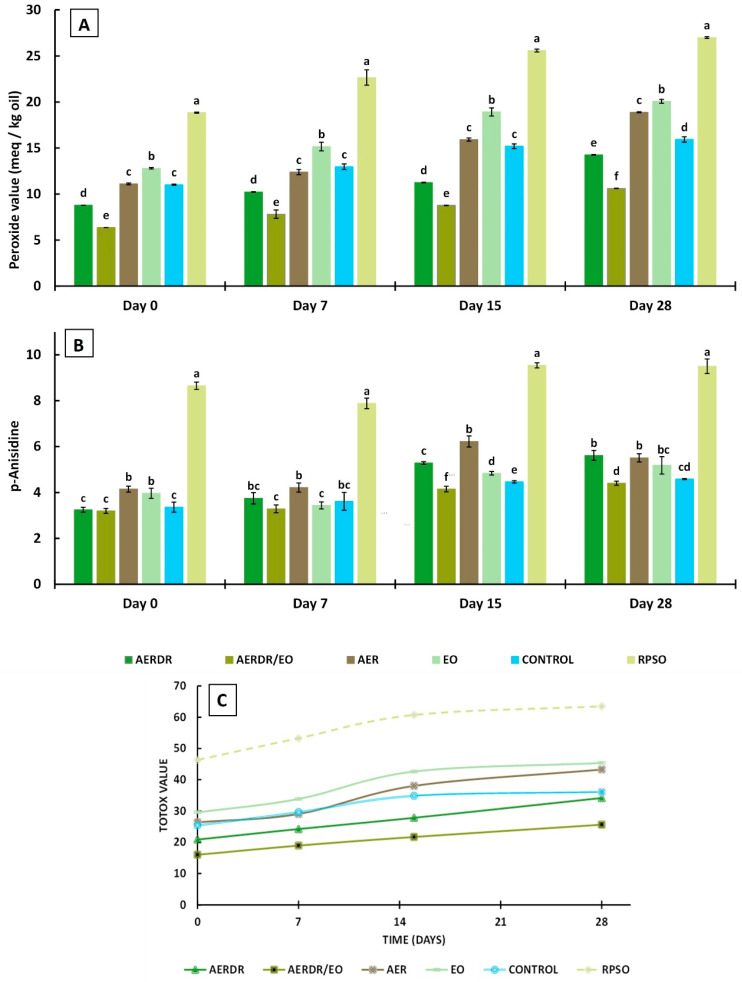
Peroxide values (**A**), p-Anisidine values (**B**), and TOTOX values (**C**) of encapsulated rapeseed oils (emulsified preparations in alginate beads) and free rapeseed oil (RPSO) when stored at 20 °C for 28 days. Data are expressed as means ± standard deviation; different letters above bars for the data sets of each specified storage time indicate significant differences (*p* < 0.05).

The PV is the most often used indicator to evaluate the state of oxidation of oils and fats. It is measured by a relatively simple assay and is especially utilized to assess oil quality after processing [59] and storage [28] or to examine the antioxidant potential of additives incorporated in oils [60,61]. The PV is determined by titration of liberated iodine, produced from the reaction of hydroperoxides with potassium iodide; hydroperoxides are formed at the early stages of lipid oxidation—the so-called “induction period/primary oxidation”—and thus, determination of PV can give an early indication of rancidity in fats and oils. In this study, the determination of the gradual progression of PVs for each type of rapeseed oil formulation (unencapsulated and encapsulated forms) with storage time (Figure 3A) implies that the rate of hydroperoxide formation is greater than their decomposition (propagation reactions) [62]. Significant differences (*p* < 0.05) in PVs of the oil samples were noted throughout storage. Over the 28-day storage, the PV value of the unencapsulated oil (RPSO) was significantly higher than the PV of all the encapsulated rapeseed oil preparations in alginate beads, indicating that the encapsulation of emulsified oil droplets in the polysaccharide matrix was effective to slow down the formation of primary oxidation products. Similarly, the encapsulation of olive oil during storage at 37 °C [28] and ginger oil during 15-day storage at 4 °C [29] using alginate and blends with other polymers were effective in slowing down the formation of primary oxidation products.

Among the encapsulated oil samples, the PVs decreased in the order of AERDR/EO <AERDR <control ≈ AER < EO and most of the PVs were below 18 meq/Kg over the storage time—indicative of acceptable oil quality. The initial PV of the encapsulated control sample was 11.02 meq/Kg and increased to 15.94 meq/Kg on Day 28, whilst the encapsulated oil with the addition of rosemary essential oil only (EO) increased from 12.8 (Day 0) to 20.7 meq/Kg by the end of storage time, suggesting that the incorporation of rosemary essential oil in the encapsulated oil did not have a positive impact against oxidative deterioration. Likewise, the encapsulated emulsified oil with the aqueous extract of phenolic compounds of rosemary raw tissues (AER) in the aqueous phase showed similar values of PVs to the control encapsulated oil sample during 28-day storage, indicating negligible ability to inhibit the generation of primary oil oxidation products. The lowest PVs were observed in the oil samples encapsulated with phenolic compounds of rosemary distillate aqueous extract (AERDR) as well as when a combination of AERDR with rosemary essential oil was used, on all days of measurements, indicating that the latter combined treatment (AERDR/EO) was the most effective to slow down the formation of primary oxidation products among all formulations tested. Additive effects between the phenols of the aqueous extract of the rosemary distillate by-products and the antioxidant compounds present in the rosemary essential oil result in greater ability to interact with free radicals, and thereby improve the protection of rapeseed oil against the undesirable oxidative deterioration. In general, the encapsulated oil samples in emulsified form (O/W) with aqueous extracts from the post-distillation rosemary residues as the continuous phase (AERDR) or in combination with EO (AERDR/EO) exhibited the lowest PVs; these observations of the oxidative stability of rapeseed oil could be attributed to the relatively high content of carnosic acid in the rosemary distillate aqueous extract, as the LC-MS analysis revealed, and is further evidenced by findings from previous studies [54,55]. Earlier works also showed that carnosic acid has a very high reactivity toward reactive oxygen species (ROS), and upon scavenging ROS, carnosic acid is converted into a variety of secondary antioxidants (such as carnosol), amplifying the antioxidant power of the precursor compound [63].

#### 3.3.2. p-Anisidine Values

**Figure 4:**
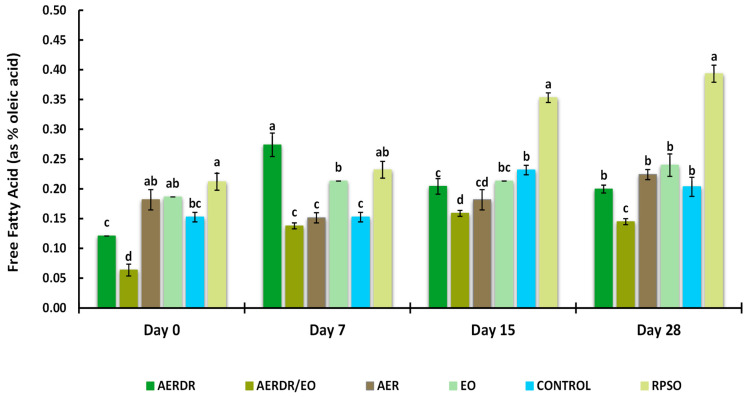
Free fatty acid (FFA) of encapsulated rapeseed oils (emulsified preparations in alginate beads) and free rapeseed oil (RPSO) when stored at 20 °C for 28 days. Data are expressed as means (wt. % of the oil) ± standard deviation; different letters above bars of each cluster indicate significant differences (*p* < 0.05).

The p-AnV index measures the secondary oxidation products, including aldehydes, ketones, and various other products. It is a useful measurement of the oxidation status of “abused” fats and serves as a good predictor of storage stability when accelerated storage tests are used [60]. Over the 28-day storage, the p-AnV value of free unencapsulated oil (RPSO) was found higher by two-fold than the encapsulated oil samples (*p* < 0.05) (Figure 4), irrespective of the day of measurement, indicating that encapsulation led to significantly lowered p-AnV values, as previously reported [28,64]. The results also revealed a gradual increase in the secondary oxidation products with the storage time for all formulations; the p-AnV values of the encapsulated oil samples ranged between 3.2 and 4.15 on Day 0 without pronounced changes on Day 7, whereas the p-AnV values were found to vary between 4.41 and 5.62 on Day 28 for these preparations. At any day of storage, small differences were noted in p-AnV values among the encapsulated oil samples, with the preparation consisting of the aqueous extract of phenolic compounds from the post-distillation rosemary residues and the rosemary essential oil (AERDR/EO) exhibiting the lowest p-AnV values (Figure 3B), in accordance with the trends of the PV values (Figure 3A). Generally, the p-AnV should be below 10 for the acceptable quality of edible oils [41]. Overall, the p-AnV values of all oil samples did not exceed this maximum threshold value for any given storage time, indicating that secondary oxidation was still in its early stages.

#### 3.3.3. TOTOX Values

The TOTOX is calculated from the obtained PV and p-AnV values of the oil for a given storage time, thus providing an overall indication of the oil oxidation status. Specifically, this value provides insight regarding the oxidation history of the oil (PV) and the potential for further deterioration (p-AnV). As a general guidance, an oil is considered of acceptable quality when the TOTOX value is less than 30 [41]. It was noticed that the TOTOX values of all samples increased as the storage proceeded (Figure 3C). In general, the encapsulated forms of rapeseed oil were found to be less susceptible to oxidation than RPSO.

After 28 days of storage at 20 °C, the TOTOX values decreased in the order of free unencapsulated oil RPSO (63.50) > EO (45.39) > AER (43.29) > control (36.12) > AERDR (34.14) > AERDR/EO (25.63). Thus, only the rapeseed oil encapsulated with phenolic compounds of the rosemary distillate aqueous extract as well as the inclusion of rosemary essential oil in the oil phase (AERDR/EO preparation) had an acceptable TOTOX value (<30) until the end of storage time. In the absence of any rosemary-derived matter, the control formulation (encapsulated oil in emulsified form with water and Tween) achieved a shelf-life of 7 days. Similarly, the encapsulated oil sample prepared by emulsification with the aqueous phenolic extract from the raw rosemary tissues (AER) also displayed a maximum shelf-life of 7 days. These observations suggest that the alginate encapsulation of emulsified rapeseed oil alone cannot prevent the oxidative deterioration of the oil and neither can the use of the aqueous extract of rosemary in the aqueous phase; instead, the combination of an aqueous extract (phenolic compounds) of post-distillation rosemary residues (water phase) along with the rosemary essential oil dissolved in the oil phase of the O/W emulsions, followed by alginate encapsulation, substantially improved rapeseed’s oil oxidative stability. These findings concur with observations made by Sun-Waterhouse and co-workers who reported greater stability of olive [28] and avocado [65] oil degradation when using alginate as an encapsulating agent in the presence of an antioxidant.

#### 3.3.4. Free Fatty Acids Content

FFAs can act as prooxidants in both bulk and emulsified oils by accelerating the decomposition rate of hydroperoxides [66]. The presence of FFAs is a result of hydrolytic rancidity caused by the reaction between water and oil. Thus, high FFA content in oils can cause further oxidation contributing to the perception of “rancidity” by the development of unpleasant taste and odour [60]. The Codex Alimentarius International Food Standards (2109) [67] set a standard of 0.3% (the relationship FFA = Acid value/1.989 was applied) for good-quality refined oils. The effect of encapsulation and the studied rosemary derivatives on FFA formation is shown in Figure 4. Over the 28-day storage time, a gradual increase in the FFA content of the RPSO sample was observed; moreover, the rate of increase in the unencapsulated oil was significantly higher (*p* < 0.05) on days 15 and 28 of measurement and found at levels above 0.3%, suggesting unacceptable quality. In contrast, the rate of FFA increase for the encapsulated oil samples was found to be much lower than RPSO, showing an FFA content of < 0.3% after 28 days of storage. Among the studied rosemary adjuncts, the preparation with AERDR/EO resulted in the lowest FFA levels across all days of measurement. The FFA content of the encapsulated oil sample with the AERDR/EO varied significantly (*p* < 0.05) from all the remaining encapsulated samples at the end of the storage period. An earlier study reported that encapsulation of avocado oil with alginate and BHT or phloridzin had a subtle additional effect on the FFA content compared with the encapsulated oil in the absence of an antioxidant [28]; however, a greater decrease in FFA content was observed when the oil was encapsulated with a combined matrix of alginate and hydroxypropyl methylcellulose (HPMC). On the other hand, encapsulation with alginate and fortification with caffeic acid of olive oil did not result in delaying the formation of FFA after 30-day storage at 20 or 37 °C [65]. That was also consistent with the study of Wang and co-workers (2013) [68], who did not find any distinct differences in suppressing hydrolytic rancidity of rapeseed oil encapsulated with alginate when the oil preparations were enriched with three different antioxidants, i.e., BHT, quercetin, and vitamin E.

#### 3.3.5. Total Extractable Phenolic Content after Storage

**Figure 5:**
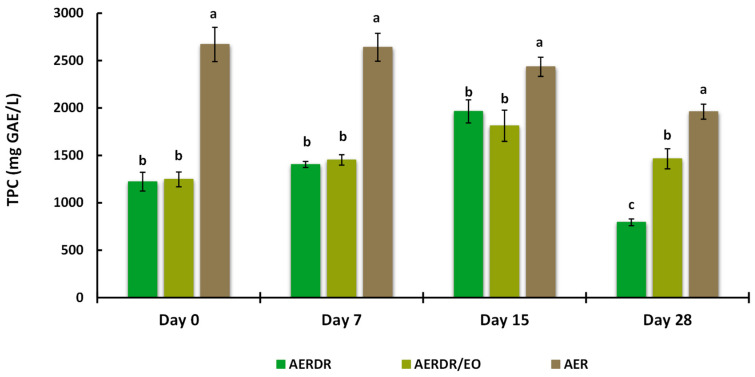
Total phenolic content (TPC) of the aqueous phase extracted from the alginate beads when stored at 20 °C for 28 days. Data are expressed as means ± standard deviation; different letters above bars of each cluster indicate significant differences (*p* < 0.05).

TEPC from the alginate beads were determined by the Folin–Ciocalteu assay and are presented in Figure 5. Significant differences (*p* < 0.05) in the TEPC content of the aqueous phase were observed between the beads made with AER and AERDR (i.e., the preparations with or without inclusion of EO in the oil phase of the O/W emulsions) over the storage time; the TEPC content of the beads treated with AER was found to be two-fold higher than AERDR, which is consistent with the original TPC results obtained for the aqueous rosemary extracts “R” and “DR” (as shown in Section 3.1). In addition, the “DR” aqueous rosemary extract, utilized in the AERDR formulation, was earlier revealed to contain a relatively high content of the hydrophobic compounds carnosol and carnosic acid; therefore, the observed difference in TEPC could be attributed to the oil extraction methods [69,70] and/or the potential partitioning of phenolic compounds in the lipid phase [71].

Regarding the beads formulated with AERDR and AERDR/EO, the TEPC of the two treatments were found at similar levels (1220–1250 mg GAE/L), a gradual increase in TEPC was observed over the first 15 days, and a noticeable decline in TEPC was finally noted on Day 28 of storage for both preparations. These results are in agreement with the study of Sun-Waterhouse et al. [28], in which olive oil (fortified with caffeic acid) extracted from sodium alginate beads showed an increasing trend of TEPC for the first 14 days and then a decline after 30 days of storage at 20 °C. In the case of the aqueous phase from the beads formulated with the AER extract, a high and constant TEPC content was revealed over the first 7 days and then TEPC also followed a downward trend until the end of the storage period. The “R” aqueous rosemary extract, utilized in the AER formulation, was earlier revealed to contain high TPC content, with rosmarinic acid as the main antioxidant in abundancy; it is hypothesized that rosmarinic acid, due to its hydrophilicity, might be predominantly bound to the alginate network (mannuronic and guluronic units of alginate) resulting in increased exposure to environmental conditions (oxygen), which subsequently might lead to accelerated antioxidant depletion and poor oxidative stability of the oil. As mentioned previously, the ability of free radical scavenging antioxidants to inhibit lipid degradation in food systems not only depends on their chemical reactivity but also on factors such as polarity, physical location, interactions with other components, and environmental conditions [72]. Thus, the antioxidant compounds present in the AER formulation might have reacted with oxygen and scavenged free radicals in the process of inhibiting lipid oxidation, resulting in levelled-off TEPC over storage time. Overall, influencing factors for the observed differences in the oxidative stability of the rapeseed oil in different alginate bead preparations could be the different total phenolic content in the aqueous extracts, exhibiting varying free radical scavenging activity [73], the polarity of the antioxidant compounds present in the extracts [74], and the physical location of the antioxidant compounds as different types of interfaces in oil-in-water emulsions can exist depending on phenolic composition [3,75]. Overall, in the present study, high amounts of phenolics were still present (extractable fraction) until the end of the stability test in all samples, implying a good retention capacity of the phenolic compounds following the encapsulation process and during storage; therefore, free phenolic compounds would be available in beads until the end of the storage period.

#### 3.3.6. Fatty Acid Composition

Fatty acid composition analysis was performed using gas chromatography to determine whether the levels of the unsaturated fatty acids are affected by the encapsulation process and the inclusion of rosemary antioxidants in the alginate beads of the emulsified rapeseed oil preparations. In total, 11 fatty acids (i.e., palmitic acid, palmitoleic acid, stearic acid, oleic acid, linoleic acid, γ-linolenic acid, cis-11-eicosenoic acid, linolenic acid, cis-11,14,17-eicosatrienoic acid, cis-5,8,11,14,17-eicosapentaenoic acid, and nervonic acid) were identified by the GC-FID method and their respective peak area percentages were calculated (Table A1). Rapeseed oil samples exhibited the typical FA profile of canola oil [76], which is mostly composed of oleic acid (C18:1) followed by linoleic acid (C18:2), corresponding to about 60% and 20% of the total FA composition, respectively.

As a general trend, oil samples, irrespective of the treatment, had shown a gradual decrease in the peak area percentage of the identified fatty acids with storage time, which is in line with previous oxidative stability studies [28,61,77]. This observation is more evident by the calculated parameters of the sum of saturated fatty acids (SFA), sum of monounsaturated fatty acids (MUFA), sum of polyunsaturated fatty acids (PUFA), and their respective ratios in the oil samples encapsulated with rosemary antioxidants (Table 3). On Day 28, the MUFA content of the encapsulated oil samples was found to be higher than in the RPSO, i.e., significantly higher MUFA content of the oil samples encapsulated with AERDR and AERDR/EO was observed when compared to the RPSO sample (*p* < 0.05). This observation was mainly attributed to the content of oleic acid (C18:1) (detailed results in Table A1). However, decreases occurred in the PUFA content in the case of encapsulated oils, whilst the PUFA content of the RPSO oil sample showed a minor increase after the 28-day storage period. The detected changes are partially in agreement with the findings of Sun-Waterhouse and co-workers (2011) [28] who reported that both encapsulation in alginate microspheres and the incorporation of caffeic acid (300 ppm) in olive oil helped with preserving the MUFA and PUFA content. Overall, the observed differences in the fatty acid profile between the encapsulated rapeseed oil samples were relatively small in the current study; elevated storage temperatures and longer storage times might reveal greater differences for losses in the mono- and polyunsaturated fatty acids in the absence of rosemary antioxidants, as the relevant literature reports also suggest [61,77,78,79]. Moreover, it has been previously described that the oxidative resistance of lipids is higher with increasing extract concentrations [77,80]. There have been several studies aiming to elucidate the potential effects of natural antioxidants on lipid oxidation and fatty acid profiles determined by gas chromatography. The study of Sun-Waterhouse et al. (2011) [28] has shown that unsaturated fatty acids of olive oil, including C18:1 (ω-9), C18:2 (ω-6), and C18:3 (ω-3), were protected by encapsulation in alginate beads and the addition of caffeic acid when stored at 20 and 37 °C for 30 days. Tohma and Turan [81] reported that hazelnut oil treated with various rosemary additives (plant, ethanolic extract, and essential oil), showed higher oxidative stability during the frying of French fries. Fhaner et al. (2016) [77] also showed that the presence of 0.84 mM phenolic diterpenes in rosemary extract preserved the eicosapentaenoic acid (EPA) and docosahexaenoic acid (DHA) levels during the storage of fish oil for 14 days at 30 and 50 °C.

## 4. Conclusions

Hydrodistillation has a strong influence on the antioxidant potency of rosemary leaves; during distillation, antioxidants are lost by the wastewater stream or thermal degradation. A solid–liquid extraction using water as a solvent and β-cyclodextrin as a co-solvent resulted in considerable amounts of phenolic compounds in the extract of undistilled rosemary leaves and the extract of post-distillation rosemary waste residues as well. The LC-MS analysis of the extracts revealed that both aqueous extracts primarily contained rosmarinic acid, salvianolic acid B, quinic acid, carnosol, and carnosic acid. The aqueous extract of raw rosemary tissues was mainly abundant in rosmarinic acid (56.60 ± 0.6 mg/g dw), and the extract of rosemary distillate residues contained both high amounts of rosmarinic acid (42.73 ± 0.28 mg/g dw) and the phenolic diterpenes of carnosol and carnosic acid (13.89 ± 0.15 mg/g dw). The differences in the polyphenols profile of the extracts may have contributed to the observed variation in oxidative stabilities among the diverse preparations of the encapsulated rapeseed oil samples in alginate beads. The encapsulation of rapeseed oil emulsions in an alginate-based gel matrix with the use of aqueous rosemary extracts, rich in phenolic compounds, has enhanced the oxidative stability of the rapeseed oil during a 28-day storage period, as monitored by the evolution of the primary and secondary products of oxidation. The alginate beads prepared with the aqueous extract of the post-distillation rosemary residues—also comprising the aqueous phase of the rapeseed oil emulsions—when combined with the rosemary essential oil dissolved in the rapeseed oil phase (AERDR/EO) exhibited the lowest lipid deterioration (PV = 10.61 ± 0.02 meq/Kg oil, p-AnV = 4.41 ± 0.09, and FFA = 0.14 ± 0.00, expressed as % oleic acid content), indicating an acceptable oil quality until the end of the storage period. The observed high protective ability of the aqueous extract of post-distillation rosemary residues in rapeseed’s oil oxidation is probably attributed to the proportionally higher amounts of carnosic acid detected in the rosemary extracts, a compound with strong antioxidant activity and known for its effectiveness in protecting vegetable oils rich in UFA from oxidation [81,82]. Moreover, high amounts of phenolics were also detected until the end of the stability tests for all rapeseed oil preparations encapsulated in alginate beads, implying a constant antioxidant environment throughout storage. Overall, the phenolic extracts obtained by applying green extraction techniques and valorizing waste residues of the essential oil industry could find applications in the food and drug industries due to their antioxidant properties and the enhanced protection they can offer for the preservation of oil-based nutraceutical formulations.

## Figures and Tables

**Table 1:** Formulation of experimental emulsions used for encapsulation of rapeseed oil in alginate beads.

Sample Identification *	AERDR	AERDR/EO	AER	EO	Control	RPSO
Rosemary raw plant aqueous extract			+			
Post-distillation rosemary residue aqueous extract	+	+				
Water				+	+	
Alginate conc. (% wt)	2.0	2.0	2.0	2.0	2.0	
Rosemary essential oil at 0.1% *w*/*w*		+		+		
Rapeseed oil	+	+	+	+	+	+
Tween 80	+	+	+	+	+	

* AERDR: aqueous extract of post-distillation rosemary waste residues; AERDR/EO: aqueous extract of post-distillation rosemary waste residues with rosemary essential oil; AER: aqueous extract of rosemary (raw plant material); EO: deionized water with rosemary essential oil; RPSO: free, non-encapsulated rapeseed oil; (+) dictates ingredients used for each formulation.

**Table 2:** Phenolic compounds identified and quantified by LC-MS in aqueous extract of dried ground rosemary (R) and aqueous extract of dried ground post-distillation rosemary residues (DR).

Chemical Group	Compound Name	Concentration (mg/g DW)	
		R ^1^	DR ^1^
Phenolic acids	Quinic acid	2.80 ± 0.07 ^a^	2.29 ± 0.06 ^b^
	Neochlorogenic acid	0.47 ± 0.02 ^a^	0.17 ± 0.01 ^b^
	Caffeic acid	0.20 ± 0.01 ^b^	0.36 ± 0.01 ^a^
	Rosmarinic acid	56.6 ± 0.6 ^a^	35.3 ± 0.5 ^b^
	Salvianolic acid B	5.13 ± 0.13 ^a^	4.61 ± 0.15 ^b^
Total Phenolic acids		65.2 ± 0.38	42.73 ± 0.28
Flavonoids	Vicenin-2	0.16 ± 0.01 ^a^	0.12 ± 0.01 ^a^
	Luteolin-7-O-rutinoside	1.73 ^a^ ± 0.09 ^a^	0.14 ± 0.00 ^b^
	Apigenin-7-O-glucoside	0.03 ± 0.002 ^a^	0.01 ± 0.002 ^b^
	Hesperidin	1.29 ± 0.075 ^a^	0.19 ± 0.01 ^b^
Total Flavonoids		3.21 ± 0.01 ^a^	0.47 ± 0.01 ^b^
Phenolic diterpenes	Carnosol	0.58 ± 0.02 ^b^	5.67 ± 0.22 ^a^
	Carnosic acid	2.51 ± 0.10 ^b^	8.22 ± 0.08 ^a^
Total Phenolic diterpenes		3.09 ± 0.08 ^b^	13.89 ± 0.15 ^a^

^1^ Mean values for each compound with different superscript letters are statistically different (*p* < 0.05); analyses were performed in duplicate.

**Table 3:** Saturated (SFA), monounsaturated (MUFA), and polyunsaturated (PUFA) proportions of fatty acids (% of total peak area in GC-FID chromatographs) as means ± standard deviations; values in the same column with different letters (a,b,c) for each storage time are significantly different (*p* < 0.05).

Storage Day	Oil Sample	SFA (%) ^1^	MUFA (%) ^1^	PUFA (%) ^1^	UFA/SFA	MUFA/SFA
Day 0	AERDR	6.14 ± 0.08 ab	61.79 ± 0.09 a	32.07 ± 0.01 b	15.44 ± 0.04 ab	10.07 ± 0.15 ab
	AERDR/EO	6.05 ± 0.02 b	61.91 ± 0.02 a	32.03 ± 0.02 b	15.52 ± 0.04 a	10.23 ± 0.03 a
	AER	6.06 ± 0.01 b	61.87 ± 0.02 a	32.07 ± 0.01 b	15.50 ± 0.03 a	10.21 ± 0.02 a
	EO	6.24 ± 0.06 a	61.18 ± 0.43 b	32.58 ± 0.42 a	15.04 ± 0.15 c	9.81 ± 0.13 c
	Control	6.14 ± 0.01 ab	61.79 ± 0.01 a	32.07 ± 0.01 b	15.28 ± 0.03 b	10.06 ± 0.02 ab
	RPSO	6.24 ± 0.02 a	61.90 ± 0.01 a	31.87 ± 0.02 b	15.03 ± 0.05 c	9.92 ± 0.03 bc
Day 7	AERDR	6.08 ± 0.01 ab	61.91 ± 0.01 a	32.01 ± 0.02 a	15.44 ± 0.04 ab	10.18 ± 0.02 a
	AERDR/EO	6.10 ± 0.02 ab	61.91 ± 0.02 a	31.99 ± 0.01 a	15.39 ± 0.04 ab	10.15 ± 0.03 a
	AER	6.10 ± 0.04 ab	61.88 ± 0.03 a	32.02 ± 0.02 a	15.40 ± 0.10 ab	10.15 ± 0.07 a
	EO	6.12 ± 0.01 a	61.92 ± 0.00 a	31.97 ± 0.01 a	15.35 ± 0.03 b	10.12 ± 0.02 a
	Control	6.12 ± 0.01 a	61.90 ± 0.01 a	31.98 ± 0.00 a	15.35 ± 0.02 b	10.12 ± 0.01 a
	RPSO	6.02 ± 0.07 b	61.48 ± 0.61 a	32.50 ± 0.68 a	15.61 ± 0.19 a	10.21 ± 0.02 a
Day 15	AERDR	6.10 ± 0.04 bc	61.84 ± 0.13 a	32.06 ± 0.09 a	15.39 ± 0.10 ab	10.14 ± 0.08 a
	AERDR/EO	6.14 ± 0.00 ab	61.80 ± 0.02 a	32.06 ± 0.02 a	15.30 ± 0.01 bc	10.07 ± 0.01 ab
	AER	6.12 ± 0.00 b	61.78 ± 0.00 a	32.09 ± 0.01 a	15.34 ± 0.01 b	10.09 ± 0.01 a
	EO	6.24 ± 0.06 a	61.18 ± 0.43 a	32.58 ± 0.42 a	15.04 ± 0.15 c	9.81 ± 0.13 c
	Control	6.23 ± 0.06 a	61.28 ± 0.42 a	32.49 ± 0.36 a	15.05 ± 0.15 c	9.83 ± 0.16 bc
	RPSO	6.01 ± 0.01 c	61.80 ± 0.01 a	32.19 ± 0.01 a	15.64 ± 0.03 a	10.29 ± 0.02 a
Day 28	AERDR	6.15 ± 0.03 ab	62.27 ± 0.01 a	31.58 ± 0.03 c	15.25 ± 0.09 bc	10.12 ± 0.05 ab
	AERDR/EO	6.07 ± 0.06 bc	62.17 ± 0.35 a	31.75 ± 0.29 bc	15.47 ± 0.15 ab	10.24 ± 0.15 a
	AER	6.11 ± 0.01 abc	61.91 ± 0.01 ab	31.97 ± 0.02 ab	15.36 ± 0.03 abc	10.13 ± 0.02 ab
	EO	6.20 ± 0.01 a	61.90 ± 0.01 ab	31.84 ± 0.02 bc	15.13 ± 0.03 c	9.99 ± 0.02 b
	Control	6.06 ± 0.03 c	62.02 ± 0.03 ab	31.92 ± 0.03 ab	15.50 ± 0.08 a	10.23 ± 0.06 a
	RPSO	6.05 ± 0.02 c	61.74 ± 0.00 b	32.21 ± 0.02 a	15.54 ± 0.05 a	10.21 ± 0.03 a

^1^ SFA represents the sum of palmitic acid (C16:0) and stearic acid (C18:0); MUFA corresponds to the sum of palmitoleic acid (C16:1), oleic acid (C18:1), eicosenoic acid (C20:1) and nervonic acid (C24:1); and PUFA represents the sum of linoleic acid (C18:2), g-linolenic acid (C18:3α), linolenic acid (C18:3γ), eicosatrienoic acid (C20:3), and eicosapentaenoic acid (C20:5).

## Data Availability

The original contributions presented in the study are included in the article, further inquiries can be directed to the corresponding author.

## Appendix B

**Table A1:** Levels of identified fatty acids (% of total fatty acid area) in encapsulated (emulsified preparations in alginate beads) and unencapsulated rapeseed oil (RPSO) samples stored for 28 days at 20 °C; comparisons of the reported mean values are made for each fatty acid separately; values in the same column with different letters (a,b,c) for each storage time are significantly different (*p* < 0.05).

Storage Day	Oil Sample	C16:0 (%)	C16:1 (%)	C18:0 (%)	C18:1 (%)	C18:2 (%)	C18:3α (%)	C18:3γ (%)	C20:1 (%)	C20:3 (%)	C20:5 (%)	C24:1 (%)	Total (%)
0	AERDR	4.56 ± 0.05 b	0.18 ± 0.03 ab	1.58 ± 0.03 abc	60.92 ± 0.16 ab	20.55 ± 0.03 ab	9.94 ± 0.01 ab	1.18 ± 0.01 b	0.56 ± 0.03 a	0.31 ± 0.03 a	0.09 ± 0.02 a	0.13 ± 0.02 a	100.00
	AERDR/EO	4.50 ± 0.01 b	0.16 ± 0.01 b	1.55 ± 0.00 bc	61.06 ± 0.02 a	20.57 ± 0.01 a	9.92 ± 0.01 c	1.18 ± 0.01 b	0.54 ± 0.01 a	0.29 ± 0.01 a	0.08 ± 0.00 a	0.15 ± 0.03 a	100.00
	AER	4.52 ± 0.01 b	0.17 ± 0.01 b	1.55 ± 0.01 c	61.04 ± 0.02 ab	20.58 ± 0.01 a	9.96 ± 0.01 a	1.17 ± 0.01 b	0.54 ± 0.01 a	0.28 ± 0.00 a	0.08 ± 0.01 a	0.12 ± 0.01 a	100.00
	H_2_O/EO	4.53 ± 0.02 b	0.17 ± 0.00 b	1.56 ± 0.00 bc	61.06 ± 0.02 a	20.56 ± 0.01 ab	9.92 ± 0.01 bc	1.17 ± 0.01 b	0.55 ± 0.01 a	0.28 ± 0.01 a	0.08 ± 0.00 a	0.12 ± 0.00 a	100.00
	Control	4.53 ± 0.01 b	0.21 ± 0.00 a	1.61 ± 0.01 a	60.86 ± 0.04 b	20.51 ± 0.03 b	9.92 ± 0.01 c	1.20 ± 0.01 a	0.57 ± 0.01 a	0.25 ± 0.16 a	0.19 ± 0.12 a	0.14 ± 0.05 a	100.00
	Rapeseed	4.64 ± 0.03 a	0.16 ± 0.01 b	1.60 ± 0.01 ab	61.04 ± 0.02 ab	20.43 ± 0.01 c	9.87 ± 0.00 d	1.18 ± 0.01 b	0.55 ± 0.01 a	0.30 ± 0.01 a	0.09 ± 0.01 a	0.13 ± 0.01 a	100.00
7	AERDR	4.52 ± 0.02 a	0.16 ± 0.00 b	1.56 ± 0.00 ab	61.09 ± 0.02 a	20.56 ± 0.02 a	9.94 ± 0.01 a	1.17 ± 0.01 a	0.53 ± 0.01 a	0.27 ± 0.00 c	0.08 ± 0.01 b	0.12 ± 0.00 a	100.00
	AERDR/EO	4.54 ± 0.01 a	0.16 ± 0.01 ab	1.56 ± 0.01 ab	61.09 ± 0.02 a	20.56 ± 0.00 a	9.91 ± 0.01 a	1.17 ± 0.01 a	0.53 ± 0.01 a	0.27 ± 0.01 bc	0.08 ± 0.01 ab	0.12 ± 0.01 a	100.00
	AER	4.52 ± 0.03 a	0.17 ± 0.01 ab	1.57 ± 0.01 ab	61.05 ± 0.05 a	20.57 ± 0.01 a	9.91 ± 0.00 a	1.18 ± 0.01 a	0.54 ± 0.01 a	0.28 ± 0.00 b	0.08 ± 0.01 ab	0.13 ± 0.01 a	100.00
	H_2_O/EO	4.54 ± 0.01 a	0.17 ± 0.00 ab	1.57 ± 0.01 a	61.07 ± 0.01 a	20.56 ± 0.01 a	9.88 ± 0.01 a	1.17 ± 0.01 a	0.54 ± 0.01 a	0.27 ± 0.01 c	0.09 ± 0.01 ab	0.13 ± 0.01 a	100.00
	Control	4.55 ± 0.00 a	0.17 ± 0.01 a	1.57 ± 0.01 ab	61.06 ± 0.01 a	20.57 ± 0.01 a	9.89 ± 0.01 a	1.17 ± 0.00 a	0.54 ± 0.01 a	0.28 ± 0.00 bc	0.09 ± 0.01 ab	0.13 ± 0.00 a	100.00
	Rapeseed	4.48 ± 0.06 a	0.17 ± 0.01 ab	1.54 ± 0.01 b	60.64 ± 0.61 a	20.48 ± 0.21 a	10.47 ± 0.89 a	1.17 ± 0.01 a	0.54 ± 0.01 a	0.30 ± 0.01 a	0.09 ± 0.01 a	0.13 ± 0.00 a	99.99
15	AERDR	4.54 ± 0.03 c	0.19 ± 0.01 ab	1.55 ± 0.00 c	60.47 ± 0.78 a	20.53 ± 0.08 b	9.91 ± 0.04 a	1.19 ± 0.01 ab	0.55 ± 0.01 a	0.32 ± 0.02 a	0.10 ± 0.01 a	0.63 ± 0.83 a	100.00
	AERDR/EO	4.58 ± 0.01 abc	0.18 ± 0.00 bc	1.56 ± 0.00 abc	60.93 ± 0.03 a	20.59 ± 0.01 ab	9.91 ± 0.01 a	1.18 ± 0.01 bc	0.55 ± 0.01 a	0.29 ± 0.00 b	0.10 ± 0.01 a	0.15 ± 0.01 a	100.00
	AER	4.56 ± 0.01 bc	0.18 ± 0.00 bc	1.56 ± 0.00 bc	60.90 ± 0.01 a	20.60 ± 0.01 ab	9.91 ± 0.01 a	1.18 ± 0.01 abc	0.55 ± 0.01 a	0.30 ± 0.01 ab	0.10 ± 0.00 a	0.15 ± 0.01 a	100.00
	H_2_O/EO	4.61 ± 0.03 ab	0.19 ± 0.01 a	1.57 ± 0.01 ab	60.29 ± 0.43 a	20.68 ± 0.12 ab	9.91 ± 0.06 a	1.21 ± 0.01 a	0.55 ± 0.01 a	0.31 ± 0.01 a	0.42 ± 0.55 a	0.15 ± 0.01 a	99.90
	Control	4.64 ± 0.04 a	0.20 ± 0.01 a	1.58 ± 0.00 a	60.38 ± 0.44 a	20.86 ± 0.22 a	9.96 ± 0.11 a	1.20 ± 0.02 a	0.55 ± 0.01 a	0.31 ± 0.02 ab	0.09 ± 0.02 a	0.16 ± 0.02 a	99.93
	Rapeseed	4.48 ± 0.01 d	0.17 ± 0.00 c	1.54 ± 0.01 d	60.98 ± 0.01 a	20.69 ± 0.01 ab	9.97 ± 0.00 a	1.17 ± 0.01 c	0.53 ± 0.00 b	0.29 ± 0.00 b	0.08 ± 0.01 a	0.13 ± 0.01 a	100.00
28	AERDR	4.59 ± 0.04 ab	0.18 ± 0.01 a	1.56 ± 0.00 ab	61.42 ± 0.02 a	20.38 ± 0.02 c	9.67 ± 0.01 d	1.17 ± 0.01 b	0.54 ± 0.01 bc	0.28 ± 0.01 ab	0.08 ± 0.01 b	0.13 ± 0.01 b	100.00
	AERDR/EO	4.51 ± 0.04 bc	0.66 ± 0.85 a	1.56 ± 0.01 bc	60.84 ± 0.49 b	20.43 ± 0.18 bc	9.80 ± 0.09 bc	1.17 ± 0.02 b	0.55 ± 0.01 ab	0.27 ± 0.01 b	0.09 ± 0.01 b	0.13 ± 0.01 b	100.00
	AER	4.55 ± 0.01 abc	0.18 ± 0.00 a	1.56 ± 0.00 bc	61.06 ± 0.02 ab	20.59 ± 0.01 ab	9.85 ± 0.01 b	1.16 ± 0.01 b	0.54 ± 0.01 abc	0.28 ± 0.00 ab	0.09 ± 0.01 ab	0.13 ± 0.00 b	100.00
	H_2_O/EO	4.61 ± 0.00 a	0.18 ± 0.01 a	1.59 ± 0.01 a	61.01 ± 0.02 ab	20.51 ± 0.01 bc	9.75 ± 0.01 cd	1.19 ± 0.00 a	0.56 ± 0.01 a	0.29 ± 0.01 ab	0.10 ± 0.00 a	0.15 ± 0.00 a	99.94
	Control	4.51 ± 0.03 bc	0.17 ± 0.01 a	1.55 ± 0.00 bc	61.19 ± 0.03 ab	20.58 ± 0.02 abc	9.80 ± 0.01 bc	1.17 ± 0.01 b	0.54 ± 0.01 bc	0.29 ± 0.01 a	0.08 ± 0.01 b	0.12 ± 0.01 b	100.00
	Rapeseed	4.51 ± 0.02 c	0.17 ± 0.01 a	1.54 ± 0.00 c	60.92 ± 0.01 ab	20.72 ± 0.01 a	9.96 ± 0.00 a	1.16 ± 0.00 b	0.52 ± 0.01 c	0.28 ± 0.01 ab	0.08 ± 0.01 b	0.12 ± 0.01 b	100.00
